# Growing Greener: Cultivating Organisational Sustainability Through Leadership Development

**DOI:** 10.3390/bs14110998

**Published:** 2024-10-26

**Authors:** Sarah Lily Resanovich, Tim Hopthrow, Georgina Randsley de Moura

**Affiliations:** School of Psychology, The Centre for the Study of Group Processes, Canterbury CT2 7NP, UK; t.hopthrow@kent.ac.uk (T.H.); g.r.de-moura@kent.ac.uk (G.R.d.M.)

**Keywords:** leadership development, environmental sustainability leadership, pro-environmental behaviours, organisational sustainability, behaviour change

## Abstract

Organisations significantly contribute to climate change, making them essential targets for climate mitigation strategies. There is an opportunity to curb organisations’ environmental impact by increasing the amount of pro-environmental behaviour (PEB) among employees. Many social and psychological factors impact an employee’s likelihood of performing PEBs. Among social–psychological factors influencing employee PEB, leadership is unique as it is a social–psychological factor that can control or influence other factors. Leadership makes performing PEBs at work and home different. Due to its unique position, leadership has garnered attention from practitioners and researchers for how it can affect organisational environmental sustainability. There is limited research focusing on how leadership development can promote employee PEB, thereby increasing organisational environmental sustainability. Researchers conducted a narrative review that provided an overview of how leadership uniquely affects employee PEB, bringing together findings from various fields. Through this review, the authors propose the ICERR model for leadership development, which outlines five key capability areas and three desired outcomes for leadership development related to environmental sustainability. This model consists of 14 proposals that provide a framework for future research and identify critical areas for leadership development programmes looking to impact environmental sustainability.

## 1. Introduction

As the effects of the climate crisis increasingly impact society, researchers and practitioners are looking for interventions and initiatives that can change behaviour. In the past years, there has been an increased focus on how organisations can curb their environmental impact and improve their environmental sustainability [1,2]. This review focuses on leadership as a key factor that warrants further investigation. The previous literature has acknowledged the significance of leadership in influencing organisational outcomes [3]. This ability to influence organisations and their employees has spurred attention from researchers encouraging exploration into environmental leadership [4]. As people across disciplines and sectors work towards generating solutions to the climate crisis—leadership remains an area for growth and opportunity for interventions. 

If organisations intend to implement environmental strategic initiatives successfully, they need individual employees’ active support and participation [5,6,7,8]. Promising findings support the notion that leaders can promote employee environmental behaviour [6,7,9], thereby creating more environmentally sustainable organisations. For the change within organisations to be successful, leadership must be developed throughout the organisation [10]. In recent years, researchers have strayed from highly individual-centric models of leadership [10,11] to an approach that supports the notion that positive qualities and skills are not innate to specific individuals but rather that development can be utilised to create better leaders who can motivate their employees. This perspective highlights a potential route to organisational environmental sustainability: leveraging leadership development to create leaders best suited to motivate employees to adopt environmental practices and engage in pro-environmental behaviours (PEBs).

Due to the multilevel nature of the climate crisis, there is a need for multi-dimensional solutions, with varied perspectives. Therefore, this review was not restricted to one subject area—it pulls knowledge from fields such as psychology, environmental science, economics, and management to create the proposed model. With a multi-disciplined approach, there is expected to be varied perspectives, focal points, theoretical approaches, methods, and assumptions [12]. These multitudes of perspectives and approaches help our conclusions be more adaptive to various settings. By creating a framework encompassing differing ontological and epistemological approaches, we attempt to mirror “real” organisational environments, which consist of many contexts and restraints and are not limited by academic disciplinary bounds.

### 1.1. Organisations and Environmental Sustainability

Right now, there is a climate crisis, and it poses a real threat to every aspect of society and the planet. This crisis can be addressed, and the effects of climate change reversed, if there is swift action at all levels of society [13]. Organisations have been called out as an area where change would make a meaningful difference. The United Nations explicitly notes that the private sector must change if the world hopes to achieve the UN’s Sustainable Development Goal to “…protect the planet from degradation…” [14]. The Intergovernmental Panel on Climate Change also called out the private sector, arguing that organisations must reduce their environmental impact now to keep warming under 2 °C [13]. There are current constraints on the private sector with competing demands for growth and profit as well as sustainability, so there is a non-trivial challenge in driving sustainable change within those constraints.

Challenges and barriers to environmental sustainability change may impede the integration of environmental sustainability into leadership development and the broader organisation. Some challenges leaders and organisations may face include the financial pressure to maximise profit in most capitalist contexts [15]. Alternatively, organisations may have the desire to invest resources into environmental sustainability. Yet, they may not be currently capable of it—this is particularly relevant for nonprofits and charity organisations that may have restricted budgets tied to government funding, grants, or philanthropy. Outside of financial pressures, there may be other resistance to environmental sustainability from individuals. For example, individual employees may not feel that environmental sustainability is relevant to the sector or important to their own work, or environmental sustainability may conflict with their current ways of working. For a more complete analysis of the barriers to employee PEB, please see a 2018 Metanalysis [16]. Social–psychological research can be particularly adept at addressing these barriers to environmental sustainability. This is because for any environmental sustainability progress to be made, some form of behaviour will have to be changed, whether it is from mass changes across individual employees or large-scale decisions from top leadership. With the framework we propose below, we take a social–psychological perspective and attempt to tackle several of the recommendations made by researchers found via the aforementioned metanalysis, including creating quality relationships, role modelling from managers, and training [16]. Challenges to environmental sustainability need to be acknowledged and explored to produce research that can adapt to challenges and practice that is effective at working within context.

Despite the challenges discussed above, the importance of changing organisational structures to become more environmentally sustainable is evident. However, the path to do so may be less clear. One identified way mentioned throughout the literature on organisational sustainability is increasing pro-environmental behaviour (PEB) among employees. Pro-environmental behaviour is any actions individuals or groups actively perform to reduce their negative impact on the built and natural world [17]. Employee PEB can vary widely, from recycling, reducing energy consumption, transportation choices, and choosing sustainable suppliers to collaborating with team members to create more sustainable internal processes. These employee PEBs can be a pathway to creating environmentally sustainable organisations.

Individual actions of employees may seem unimportant in the fight against climate change, but when behaviour change is widespread, individual behaviours cumulatively come together to create more environmentally sustainable organisations [5,16,18]. The performance of PEBs by employees can ensure the success of environmental initiatives [19] and positively impact the organisation’s overall environmental sustainability [7]. Increasing employee PEB is central to mitigating an organisation’s environmental impact, and leadership can influence employee PEB. Therefore, leadership development could create leaders who are better situated to increase employee PEB, subsequently increasing the overall organisational environmental sustainability.

### 1.2. Aims of Present Review

This narrative review aims to contribute to our understanding of leadership development’s role in creating environmentally sustainable organisations. There has been increasing research on leadership and its relation to environmental sustainability in recent years. Much of the research is focused on how leadership, in conjunction with various factors, can promote (or hinder) employee PEB. Researchers have taken a variety of approaches and looked at many different factors. For example, researchers in Thailand have explored how leadership and spirituality can affect pro-environmental behaviour [20]. Other researchers have conducted studies in China that explored green dedication for explanations of the relationship between leadership and voluntary pro-environmental behaviour [21]. Another example is research conducted in Nigeria, which supports most of the literature and illustrates a significant relationship between environmentally specific transformational leadership and green employee behaviour [22]. This research also expanded upon findings by uncovering environmental concern as a significant mediator between environmentally specific transformational leadership and green employee behaviour [22]. These examples demonstrate some of the many research articles published in recent years exploring how leadership impacts employee environmental behaviour.

Despite this expanded focus on sustainability within leadership research, there is still relatively limited research on how leadership development plays a role in a leader’s ability to increase employee PEB and improve organisational environmental sustainability. In this review, we address this gap in the research by gathering evidence surrounding the development of environmentally sustainable leadership. We also reviewed research on leadership’s impact on environmental sustainability to see what themes frequently occur in the literature. We then highlight these themes within a proposed model to provide insights for leadership development researchers and practitioners on what key capabilities are needed to develop leaders best situated to promote PEB and create more environmentally sustainable organisations. By doing this, we aim to set a research agenda for expanding leadership development research focusing on environmental sustainability. We also encourage researchers to continue exploring how leadership can impact organisational environmental sustainability and begin expanding the research into how leadership development can better situate leaders to promote organisational sustainability.

This narrative review starts by reviewing the literature surrounding PEB in the workplace versus the household and concluding that leadership makes the difference between performing PEBs at home and in the workplace. We explain why leaders are crucial in environmental sustainability efforts and the social and psychological factors that impact leadership’s effectiveness in promoting environmental sustainability efforts through employee PEB. Then, we propose a framework of key capabilities that leaders can develop to increase their ability to encourage PEBs among employees. After establishing this framework, this review will outline how these concepts can provide a springboard for future research and how practitioners can implement changes to their leadership development programmes to include the key capability areas of the framework.

Our framework establishes a starting place for our understanding of how leadership development can impact organisational sustainability. Embedded within the framework are 14 propositions—these are offered by the authors based on conclusions drawn from previous work on leadership and environmental sustainability. These propositions provide a structured path to launching research into leadership development and environmental sustainability. Future research can strengthen, change, and add to the model as we continue to understand leadership and environmental sustainability better.

## 2. Methods

We started the literature review through Scopus, APAPsychNet, and Google Scholar. Several rounds of searching keywords related to the topic, such as leadership development, environmental leadership, and employee pro-environmental behaviour, resulted in 776 papers being screened for relevancy based on their title and a short description. After that review, 203 article abstracts were screened for relevance. Full articles were reviewed if their abstract contained information about successful environmental sustainability leadership, leadership development principles, leadership’s relationship with PEB, or leadership development in relation to behavioural change. Articles, conference papers, pre-publishes, and dissertations were included in the literature search. Articles were excluded if they were not written in English. In addition to the papers found in the initial search, many resources were found through ‘snowballing’, with sources found because they were either cited by documents from the initial search or cited those papers. Some papers were also reviewed based on suggestions from colleagues and peer reviewers.

**Figure 1:**
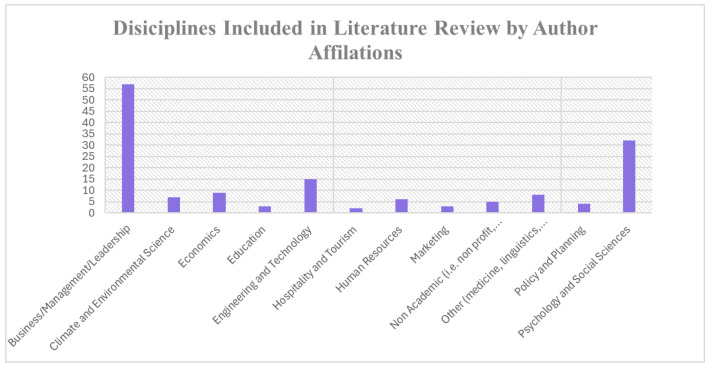
Disciplines included by authorship.

When papers were read, notes were taken on the article’s main points and how the work related to the development of environmentally sustainable leadership. We noted re-occurring leadership capabilities and themes mentioned in previous papers that were related to or suggested as components of leadership that impact PEB. After initial reading and review, areas of focus, which later became five leadership capabilities in the ICEER model, emerged. These then became the focus of the literature review. After conducting the primary search, which informed the initial structuring of the paper, five smaller searches were conducted within each competency area within the proposed model. Any articles deemed relevant were stored in Mendeley. The final iteration of this narrative review encompasses 86 papers and includes papers from a variety of disciplines. See Figure 1 for a breakdown of papers included by discipline. Papers were included in the final review if they were relevant to developing environmental sustainability leadership or the key competencies proposed to increase organisational environmental sustainability through leadership.

*If papers were authored by multiple authors representing different departments and disciplines, the papers will be included in the counts of all relevant disciplines. For a paper with three co-authors, one from the business school and two from the department of psychology, the paper would be included to add one to the total for both the department of psychology and the business school.

**For clarity, we have combined some disciplines into larger overarching groups—e.g., researchers from management departments and business schools were grouped together because of the similarities in approaches, perspectives, and methods used.

**Figure 2:**
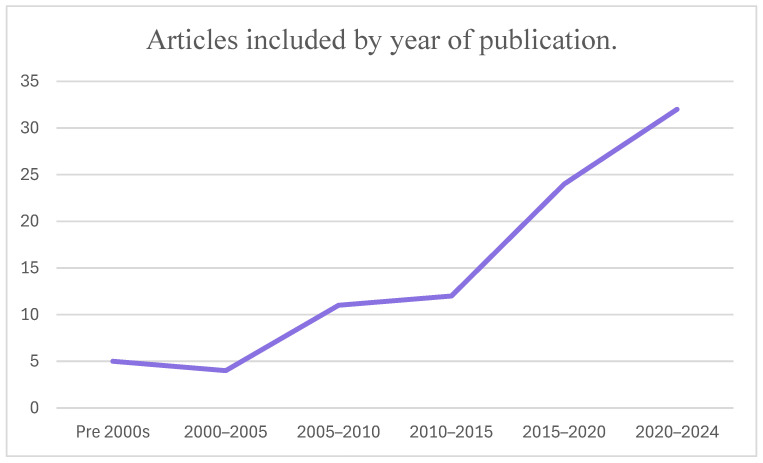
Articles included by year of publication.

There has been an increase in research into environmental sustainability in recent years, but within the last decade, there has also been a strong increase in environmental sustainability leadership research. A total of 64 percent (56 papers) of the sources in this review were published in the last ten years. Furthermore, 32 of those papers were published since 2020, demonstrating the clear interest in these issues among researchers. See Figure 2 for a breakdown of articles included in this review by publication date.

**Figure 3:**
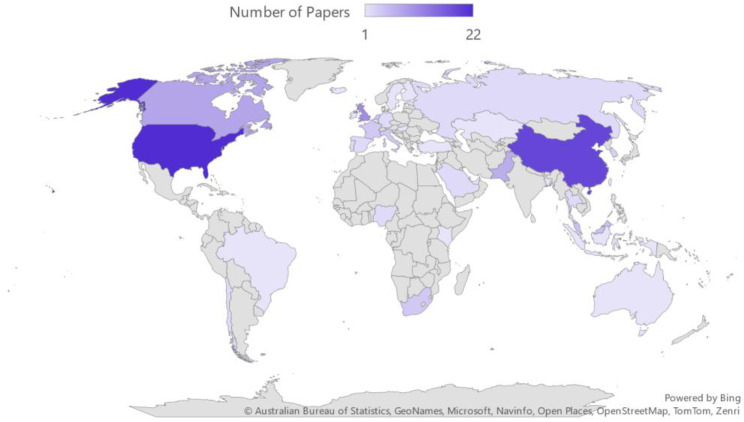
Authorship included in review by country.

**Figure 4:**
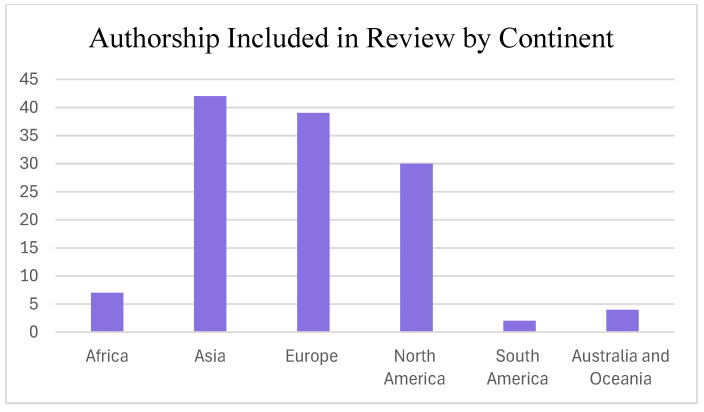
Authorship included in review by continent.

Due to the global nature of the climate crisis, there is interest in environmentally sustainable solutions globally. Therefore, a geographically diverse range of voices must be included in discussions. To increase diversity, it is helpful for researchers to clearly state the geographic spread of where the research within the paper is being generated. By doing so, we can acknowledge our shortcomings and strive to include research from various global perspectives. See Figure 3 and Figure 4 for a breakdown of papers contained within this review by country and continent.

Western countries like the United States still comprise a large share of the research in our review (22 papers included in the review had authors from American institutions). However, Asian countries (particularly China, which is represented in 19 papers) make up the largest share of papers by continent. Most of the sources contributing to this review are from Asia, Europe, and North America. Future work on leadership development and environmental sustainability should strive to include more African, Australian, and South American research.

Although including various geographically diverse voices is important in any research, it is particularly crucial in climate and environmental research as climate solutions must be applied or adaptable to a wide range of communities and cultures. Although we believe we were successful in integrating research from a variety of countries, more geographic diversity could strengthen the proposed model and ideas discussed here in the future.

## 3. Results

### 3.1. Social–Psychological Factors That Influence Employee PEB

Various factors can broadly affect whether an employee performs PEBs. These factors can be categorised into psychological or internal factors and social or external factors [23]. Internal factors explored in the literature include norms [23,24,25], personal environmental values [26], and perceived locus of control [27]. Examples of external factors include leadership [8], infrastructure, rules, and regulations [28]. Both psychological and social factors impact how much PEB is performed in the workplace. There are also several cases where these factors mediate and moderate each other (examples include [29,30,31,32]). One specific example includes a survey of leaders and employees at universities and hospitals in Pakistan conducted in three waves [32]. Among other findings, they found that the relationship between ethical leadership and employee green behaviour is sequentially mediated by green psychological climate and employee’s harmonious environmental passion [32].

Another example comes from researchers who tried to address the mixed findings on the relationship between environmental intentions and behaviour [31]. They surveyed nurses’ green behavioural intentions by asking items such as “I intend to perform pro-environmental behaviours while at work” and employee green behaviour through measures such as “Thinking about your work today, to what extent did you avoid waste?” along with questions aimed to assess ethical leadership perceived by the nurses [31]. Their data analysis revealed that the relationship between intentions and behaviour is moderated by ethical leadership [31]. This and the paper discussed above are just two examples of cases where employee PEB was explained through different moderation and mediation relationships. Various mediation and moderation effects in the existing literature reveal complexities that demand a nuanced approach to understanding employee PEB.

Much research into social–psychological factors affecting PEB has been conducted in households. Due to nuances between household and workplace settings, behavioural interventions must be tailored to the specific situation [28], and findings in household settings are not always transferable to the workplace [33]. Although, researchers can look to studies conducted in non-workplace settings to learn from findings and gain ideas for workplace-based research.

One area where household and workplace settings may differ are on the effect of values. Research findings are mixed on whether personal environmental values play a strong role in employee PEB. Some research found relationships between personal environmental values and workplace PEB through motivation [26], while other authors suggest that at work, situational factors may prevent individual employees from acting consistently in line with their personal values and norms [28].

Beyond personal values, external factors can also differ between the workplace and household settings. Many employees have limited to no control over their office infrastructure, availability of recycling bins, how the thermostat is controlled, etc. Past research has found that when there are fewer obstacles in the way of performing PEBs, individuals are more likely to perform PEBs because it becomes easier [34]. From this, it can be predicted that if there are more physical environmental obstacles in the way of PEBs, employees may be less likely to perform them even if they would perform a comparable PEB at home.

Cost is another external factor that exists in both workplace and household settings and can be explored more in research. Cost is a factor that can function differently in the household versus the workplace. In the household, finances may act as a direct external factor that could influence decision-making for individuals. In the workplace, employees, particularly those in front-line positions, may not be the ones making financial decisions. Therefore, they might not be as motivated by finances when making PEB-related decisions because the finances are not their own; instead, they are the company’s assets.

Internal and external factors interact within the workplace, making it unique from other settings. As discussed above, there may be constraints at work that prevent employees from performing the same PEBs they perform at home. Factors exist within the workplace that may change the salience of personal values, such as perceived organisational support [35], obstacles within the organisational infrastructure [34], and managerial infrastructure, e.g., if the human resources department engages with Green Human Resources Management (GHRM) principals [36]. These various social and psychological factors have a role in determining how much PEB employees perform.

### 3.2. Why Leaders Are Uniquely Situated to Increase Employee PEB and Impact Organisational Sustainability

Leadership exists formally and informally at many levels of an organisation, from line managers to the C-suite, and it is an important differentiator between workplace and household settings. Leadership is a factor in its own right—as seen by studies investigating leadership support [23] and leadership behaviour [8]. However, leaders also can control other factors [23]. For example, they can increase the number of recycling bins in the office or send their employees for environmental sustainability training. Leaders are decision-makers. They set budgets, support employees, set organisation-wide goals, and create a strategic vision. All these functions can impact employee PEB.

Leaders can remove situational barriers which positively correlate with employee PEB [34]. Leaders can also go beyond that, as those highly skilled at inspiring and motivating their employees can demonstrate their passion and influence employees’ psychological state, encouraging them to move past perceived and concrete obstacles [6]. Leaders can make an impact on employees not only by removing physical and psychological impediments to PEB but also by empowering their employees to overcome barriers that cannot be removed due to situational factors. However, organisations cannot assume that leaders will naturally be able to take on this role, as even strong leaders will not naturally be skilled at promoting change, leading transformations [37], or other skills to increase employee PEB. Therefore, leaders require development to be better suited to removing barriers and promoting PEB among employees.

### 3.3. Leadership Development: A Key to Unlocking Leadership’s Environmental Capabilities

Past research has highlighted that supervisory support is an important factor in environmental sustainability within organisations [1,19,38]. Even when research did not establish a direct link between perceived supervisory support and PEB, they found other ways leadership could influence employees, such as through leader’s behaviours [8]. Various studies have also connected different leadership styles to increased employee PEB [7,39]. The relationship between leadership and PEB is complicated and contextually dependent. Potentially, these complications are why leadership is underutilised (compared to other strategies) in its ability to affect employee PEB and organisational sustainability.

Leadership has the potential to make or break an organisation’s success when it comes to environmental sustainability efforts. So, implementing interventions that leverage leadership’s position to contribute to organisational sustainability should be an area of concern for researchers and practitioners. Leadership and leadership development are both fields spanning decades of research, but only in relatively recent years have researchers responded to the environmental challenges and begun to research leadership’s relationship with environmental sustainability and PEB. Robertson and Barling looked at the concept of environmentally specific transformational leadership starting in 2013, finding that this style of leadership had a relationship with employee PEB through the employee’s harmonious environmental passion and that the leader’s behaviours had both direct and indirect relationships with employee PEB [6]. Their research lays out many additional paths to explore leadership and PEB.

In addition to the broader research into transformational environmental leadership, there have also been explorations into the effects of other leadership styles. In 2024, a paper looked at a contrasting leadership style, transactional leadership, and its relationship with employee green creative behaviour in the Chinese tourism industry [40]. Their results revealed many direct and indirect relationships, including a positive relationship between transactional leadership and green creative behaviour among employees [40] This and the example above are just two explorations of the impact of different leadership styles on PEB.

A growing body of literature on the relationship between leadership and sustainability exists. However, the research on developing leaders who can create more environmentally sustainable organisations is sparse. One paper recently tried to address this gap by exploring if environmental transformational leadership can be developed through leadership training [41]. Nduneseokwu and Harder’s study showed promising results, with employee PEB ratings increasing after their leadership training intervention [41]. The intervention consisted of two stages. The first stage was modelled after the WeValue InSitu approach, which has been demonstrated to prompt transformational learning [42] and facilitate the transformation of tacit knowledge to explicit knowledge [41]. In this first stage, participants are exposed to photo elicitation and trigger statements to share their tacit knowledge. Then, they discuss their experiences and negotiate which of the aspects of their experiences are important to the group. After this pre-session, participants moved on to a training on environmental transformational leadership. This main training included a facilitated reflective exercise, education on the Full Range Leadership model (highlighting transformational leadership), practical exercises to discuss examples of different leadership styles, an environmental documentary, and a goal-based task where participants created specific goals for incorporating environmental transformational leadership into their work [41]. Nduneseokwu and Harder’s 2023 work demonstrate that environmental transformational leadership can be practically developed [41]. Despite this promising finding, a better understanding of the underlying mechanisms of the training that generated these positive results is needed. A gap exists in understanding how leadership development can increase employee PEBs through leaders. One approach to this could be by identifying key capabilities needed for successful environmental sustainability leadership development and testing those capabilities theoretically and practically.

### 3.4. Moving Away from the Leadership Style Categorisation Approach

Previous research has been primarily tied to individual leadership styles such as transformational leadership, e.g., [43], transactional leadership e.g., [40], and ethical leadership e.g., [44]. The previous literature has even broken down these categories further to create environmental-specific leadership style categories. See concepts such as environmentally specific transformational leadership e.g., [6,39,41] and environmental servant leadership, e.g., [20,45,46]. Researchers seeking to understand mechanisms underlying leadership’s relationship with employee PEB may miss the intricacies within the relationship because leadership styles can silo research. It also may make it harder for practitioners to navigate the literature to create research-backed interventions if they must sort through leadership typology jargon. Therefore, this review combines research from various leadership styles into one leadership development framework—ensuring that organisations can utilise findings across leadership styles. Although, this review includes articles grounded in specific styles, it will not endorse one as the best—this is to prevent reliance on one leadership style to ‘fix’ environmental sustainability problems within organisations.

Despite past research linking the influential nature of leadership behaviour to specific leadership styles, arguably, most leaders of any style can use their behaviour and influence to impact employees’ attitudes and behaviours. Most leaders do not adhere strictly to one leadership style over the other. Indeed, some even argue that leaders must utilise multiple leadership styles to succeed in the modern business world [47]. Leadership development programmes should aim to take findings across the literature and build leaders that can better pivot from one skill (that may be based on one leadership style, e.g., transformational leadership) to another skill based on another leadership style, e.g., ethical leadership. Creating leaders who can utilise strengths from various styles can be accomplished by using adaptive frameworks.

### 3.5. Important Factors for Leadership Development: The Proposed ICEER Model

**Figure 5:**
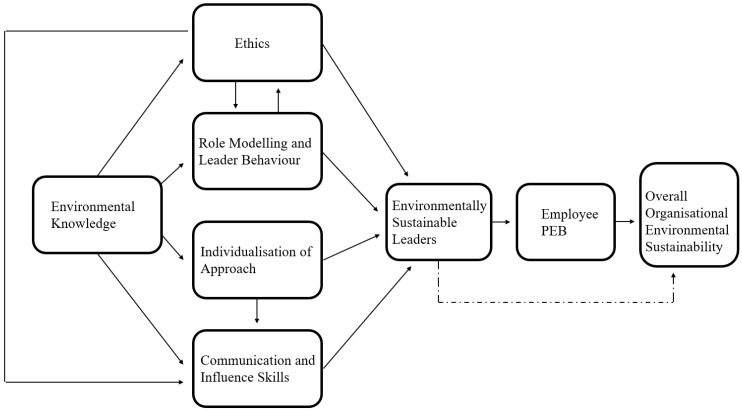
Proposed ICEER model for environmental sustainability leadership development.

We reviewed the literature to posit a leadership development model highlighting key capabilities that need to be developed to increase a leader’s ability to improve organisational sustainability through increasing employee PEBs. We propose the “ICEER” model, which provides a framework consisting of five key capability areas and three desired outcomes. The key capability areas are environmental knowledge, ethics, role modelling, individualisation of approach, and communication and influence skills. The three desired outcomes are environmentally sustainable leaders, employee PEB, and overall organisational environmental sustainability. See Figure 5 for the full ICEER Model.

Leadership development adhering to this proposed model can develop and promote these key attributes and factors to increase employee PEB and improve overall organisational sustainability. Factors within the ICEER Model are interdependent—although integrating a focus on one or two of these factors may help increase employee PEB through leadership development; approaches that incorporate the whole model and acknowledge that interconnectedness are most likely to result in the best organisational environmental sustainability.

At the end of each section, we provide propositions to outline takeaways from the ICEER model. These propositions represent the key proposed tenets of the ICEER model that form the structure of the model. Each proposition conveys a relationship that is hypothesised via the model, based on the findings of previous research. Propositions are provided to clearly distinguish the different relationships within the ICEER model to the reader and provide testable hypotheses for future research.

### 3.6. ICEER Model: Outcome Variables

Organisations need metrics to understand if their environmental sustainability initiatives are working. Therefore, when we created this model, we knew we needed to include measurable outcome variables. The three desired outcomes are interconnected: environmentally sustainable leaders, increased employee PEB, and increased organisational environmental sustainability. The five key capabilities within the proposed model impact environmentally sustainable leadership directly and indirectly. After leadership development focusing on these key capabilities, leaders should be better able to encourage employee PEB. As discussed earlier in this review, employee PEB is key to the success of any organisational environmental sustainability initiatives [5,16,18,19].

Employee PEB can be influenced by leadership and measured, making it a key outcome variable within the model. However, leaders are not limited to increasing overall organisational environmental sustainability through employee PEB, so the ICEER model proposes that they can also have a direct effect. Depending on their position within the organisation (e.g., c-suite vs. middle manager), leaders have more power than the average employee. This power allows them to make certain decisions and changes that could directly impact organisational environmental sustainability.

#### Measuring the Outcome Variables

Environmental sustainability leadership can be measured in many ways, and an organisation’s choice of measuring it should be grounded in the given context. One way to measure it would be to survey employees’ perceptions of how environmentally sustainable their managers are. Organisations could survey their employees by adapting the environment transformational leadership scale developed by [48], which was recently found to have high reliability (Cronbach alpha between 0.93 and 0.98, [41]). Measuring the levels of environmental sustainability leadership through the employees’ views is critical because, as will be discussed below, the perceptions of leaders’ impact the leaders’ ability to influence employee PEB.

The second outcome variable, employee PEB, can be measured in several ways, the first being employee self-report. However, many researchers argue the risks of measuring behaviour solely through self-report scales, especially regarding validity concerns [49]. Therefore, it is best if organisations can implement physical measures of employee PEB (this can also be accomplished in tandem with self-reporting). Physical measures of employee PEB can range from power and water usage data to waste audits to the number of individual teams pivoting from more resource-intensive processes in favour of more environmentally sustainable ones. Physical measures of PEB are present in the existing literature; examples include researchers measuring energy consumption within different teams in a hospital [50] or researchers who looked at how sustainable building design can impact individuals’ environmental behaviour through the observation of waste disposal practices [51]. In the practitioner context, environmental teams, HR, facilities teams, and senior management must collaborate to devise specific measures to capture the employee PEB they want to increase within their organisation.

Finally, the question of how to measure an organisation’s overall environmental sustainability can be resolved by creating challenging and clear environmental sustainability goals. Goals allow organisations to benchmark their progress and have a clear path towards environmental sustainability. By publicly announcing these goals, organisations will be accountable to their employees, the public, and any relevant regulators. To measure overall organisational environmental sustainability in research contexts, researchers can look at both physical metrics (e.g., resource usage) and perceptions (of the public or of employees).

**Proposition** **1:** environmentally sustainable leaders will be better situated to increase employee PEB;

**Proposition** **2:** increasing employee PEB will lead to increased overall organisational environmental sustainability;

**Proposition** **3:** environmentally sustainable leaders can directly impact overall organisational environmental sustainability through high-level decision-making processes and the creation of environmental sustainability initiatives.

### 3.7. Environmental Knowledge and Skills: Strengthen Capabilities Through Knowledge

Research has found positive effects from environmental or ‘green’ training. A study in the Italian healthcare sector explored the effects of the implementation of ‘green’ training programs on the PEBs among a variety of hospital staff members ranging from doctors to administrative staff. The results showed a positive relationship between ‘green’ training and individual employees’ PEB [52]. This study also showed a promising side benefit of green training—a positive association with job satisfaction [52]. If environmental training can increase employees’ PEBs, then it can help improve the number of PEBs a leader performs and most likely increase their ability to pass it along to their employees. For other examples of research that explores the effect of environmental or ‘green’ training on employee outcomes, see a 2022 study, which examines how training (in addition to several other GHRM factors) influences students in Northern Cyprus as prospective employees [53]. They concluded that organisations should engage in environmental education training to teach employees the importance of sustainability [53]. Another study looked at employees at 30 different universities in Kenya. Their findings suggest that training programmes centred around environmental initiatives may positively affect employee PEB [54].

By applying the findings above and other research that demonstrates the positive effects of environmental training on leadership development, the argument for incorporating environmental knowledge training is evident. Integrating environmental knowledge training into leadership development will give leaders the environmental knowledge they need to leverage the other key capabilities within the ICEER model and encourage employee PEB throughout the organisation. Leaders will also be better qualified to train their employees on environmental knowledge and pass their findings along.

#### Acknowledging the Limits of Education: Why Environmental Knowledge’s Impact Is Moderated by Social–Psychological Factors

The ICEER model incorporates education because of the promising findings of green training discussed above. Incorporating environmental education into leadership development is important because although education alone may not have a causal relationship with behaviour, a lack of knowledge is a barrier to desired behaviours [55]. The other development areas within the ICEER model complement the knowledge gained from environmental training.

Many traditional approaches to promoting PEB centre around the knowledge deficit model, which implies that individuals will change their opinions and behaviours when provided with sufficient information about a specific topic [55,56]. PEB policies and campaigns based on the knowledge deficit model aim to inform the public of the specifics of the climate crisis and the science behind climate change. However, the knowledge deficit model is highly criticised for being too simplistic [57] and for not always accurately predicting behaviour. This gap between education and behaviour could be partially because knowledge can be misunderstood, misused, or inherently flawed because the people who generate and consume the knowledge are flawed [58]. Despite many criticisms, the knowledge deficit model remains central in many climate change efforts, which can be detrimental. The knowledge deficit model assumes that knowledge exists in a pure objective form [58], failing to acknowledge nuances and realities of socio-political–environmental challenges.

Our proposed model responds to the criticisms of the knowledge deficit model by not viewing the relationship between knowledge and behaviour as linear but instead placing the relationship between knowledge and behaviour within a social–psychological context. The social-psychological context acknowledges that relationships and their socio-psychological antecedents influence how knowledge is transferred and acted upon. Many studies show that although education is a critical prerequisite for PEB, education alone is not enough to encourage individuals to perform PEBs [59]. We follow findings that have shown how viewing environmental education with a social–psychological perspective is useful in PEB research [59]. When understanding PEB, it is essential to acknowledge environmental education’s role. However, it is equally vital to understand education’s limitations without further behavioural interventions or acknowledgements of the social–psychological factors that impact how knowledge is transferred and understood. This limitation is why the ICEER model proposes that although education is necessary, its effect must be moderated through social–psychological factors because it is not enough on its own.

**Proposition** **4:** environmental knowledge is a key prerequisite for developing effective environmental sustainability leaders;

**Proposition** **5:** ethics, role modelling, individualisation of approach, and communication and influence skills will moderate the effectiveness of integrating environmental knowledge into environmental leadership development.

### 3.8. Ethical Leadership: Develop Leaders Better Positioned to Promote Behaviour

Researchers define ethical leadership as “the demonstration of normatively appropriate conduct through personal actions, interpersonal relationships, and the promotion of such conduct through two-way communication, reinforcement, and decision-making” [60] (p. 120). Research has demonstrated that developing ethical leadership is important because it can have a positive impact on desired organisational outcomes [61]. Previous qualitative research interviewed ethics officers and executive leaders, finding that ethical leaders were thought to be honest, trustworthy, and people-oriented [62]. The perception of leaders matters because their behaviours impact employee PEB [8], and the perception affects how they can or cannot influence their employees.

Leadership training must convey the importance of ethics and give leaders an understanding of the ethical dilemmas they may face in the workplace. By incorporating ethics into leadership development, organisations can increase employees’ perspectives of the trustworthiness of their leaders. Past research has found a positive relationship between ethical leadership and trust [60,62,63,64]. Ethics complements many aspects of the ICEER model, but the importance of ethics is particularly highlighted in role modelling and communication and influence skills. The qualitative study mentioned above found that ethical leaders were perceived as role models [62]. This finding indicates that developing ethical leaders could help prime leaders to be viewed as role models. With additional training focused on environmental role modelling and the impact of their own behaviours, leaders can increase their employee’s PEB through their own actions.

Ethics has been linked to both pro-social behaviour [60] and pro-environmental behaviours (see [31] for an example in the healthcare context and [44] for an example from the banking sector). Past articles point back to social learning theory [60,65] and social exchange theory [66] to explain why ethical leaders impact their followers’ behaviour. Brown et al. base their 2005 conceptualisation of ethical leadership on social learning theory, arguing that a component of ethical leadership is creating and modelling a fair system of positive and negative outcomes for normatively appropriate and inappropriate behaviour [60]. Ethical leadership can trigger social learning processes that could increase desired behaviours [60]. In the case of PEB, this research suggests that ethical leaders will be better positioned to act as role models, which, as it will be discussed, is important in promoting employee PEB.

In addition to perspectives on social learning processes and how they relate to ethical leadership, social exchange theory can also explain why ethical leadership can be leveraged to increase PEBs across organisations. Social exchange theory theorises that relationships operate according to norms of reciprocity [66,67]. From a social exchange theory perspective, when an employee feels that they have been appropriately treated by their leader or their organisation, then they will reciprocate [66]. By applying the social exchange theory perspective to the context of PEB, it can be predicted that ethical leaders can increase employee PEB by establishing positive relationships with their employees, making it more likely for the employee to reciprocate positively through performing duties beyond their job—which could include PEBs.

The definition of ethics used above highlights the importance of norms within our conceptualisation of ethics [60]. This definition allows for an interpretation of what is and is not normative conduct within a given context. Within organisations, several levels of norms could impact the view of ethical leaders, e.g., cultural and national norms, organisational level norms, or team/department level norms. Therefore, what is perceived as ethical leadership may largely depend on the follower’s perspectives on normative conduct [60]. Therefore, leadership development should aim to make leaders aware of ethical norms and the perspectives of their employees. Overall, the demonstrated connection between ethical leadership and pro-social behaviour and, more specifically, PEB points to the importance of integrating ethics into leadership development frameworks looking to increase PEBs.

**Proposition** **6:** developing ethical leadership will create leaders better able to promote PEB;

**Proposition** **7:** by developing ethical leadership, development programs will make leaders more effective at role modelling PEB;

**Proposition** **8:** by developing ethical leadership, development programs will position leaders better to communicate the environmental sustainability goals of an organisation and influence employees to contribute to those goals.

### 3.9. Leadership Behaviour: Developing Role Models for Environmental Sustainability

Leaders are a part of the landscape of an organisational environment. Field theory asserts that employees’ reactions within the environment (which in this case would be their decision to perform or not to perform PEBs) are determined by the proximity and salience of the different parts of the workplace landscape [19,68]. From individual employees’ perspectives, their direct managers may be some of the most salient and proximal individuals within their work life [19]; therefore, it can be predicted leadership behaviour holds a special role in influencing their behaviour. This extra attention on leaders allows them to act as role models and influence their employees’ behaviour by leading by example. Leadership development programmes must prepare environmental leaders to leverage their behaviour and become environmental role models who act as stewards for environmental sustainability initiatives across the organisation.

There is evidence in the literature that the PEBs that leaders do (or do not) perform impact on how much PEB their employees perform [9]. Leadership behaviour can act as a bridge between employees and the organisation—impacting the rates of employee PEB [69]. A group of researchers surveyed employees at four different housing associations in the Netherlands and found significant correlations between behaviours leader’s behaviour, employees’ intention to act, and PEB [8]. Their findings supported suggestions from Paillé and Boiral’s 2013 article that used the social exchange theory to explain organisational citizenship behaviours for the environment’s (OCB-E) determinants at work [70]. In that paper, they suggest strategies to increase PEB, which could include showcasing the commitment of managers to environmental sustainability [70]. Recent studies have also supported the positive relationship between leadership behaviour and employee PEB. One example is a cross-sectional survey conducted in 2019 to determine relationships between social–psychological factors and employee PEB among hotel workers in South Africa [71]. Additionally, other researchers argued for the importance of role modelling within their definition of environmentally specific transformational leadership—highlighting that leaders exert idealised influence by acting as environmental role models [7].

As mentioned in the section covering the impact of ethics in promoting PEB, a relationship exists between how ethical a leader is perceived and how effective of a role model they are. This is a two-way relationship where (1) to motivate employees through role modelling, the leader must be perceived as ethical and credible, and (2) to be perceived as ethical, employees must see their leaders engaged in normatively appropriate conduct [60]. This demonstrates the interconnectedness between key capabilities within the ICEER framework, particularly when it comes to the effectiveness of leadership behaviour as a motivator for PEB. Research shows the importance of role modelling and leadership behaviour in encouraging PEB, so development must not only prepare leaders to be role models but also develop leaders in areas such as ethical leadership as that will determine the effectiveness of their role modelling.

**Proposition** **9:** leadership behaviour and role modelling impact employee PEB. Leadership development that makes leaders aware of their position as role models and teaches them how to leverage that will create leaders better situated to increase employee PEB;

**Proposition** **10:** how leaders behave will affect how ethically they are perceived. This will impact their ability to influence environmental behaviour among employees.

### 3.10. Individualisation of Approach: Develop Leaders Who Can Leverage Diverse Perspectives

The challenges posed by the climate crisis are too multifaced and complicated for any one person to tackle alone [72]—which is why the ICEER model focuses on developing leaders best situated to influence all their employees to take action, perform PEBs, and contribute to increasing the overall organisational environmental sustainability. Employees come into the workplace with their own attitudes, skill sets, identities, environmental awareness, morals, and beliefs. These are just some of the psychological factors that affect an employee’s level of PEB. As stated above, there has been much research into the different factors that influence employee PEB, see [8,23,26,28,73]. Leaders need to understand their employees’ differences and tailor their approach to each employee accordingly. Effective sustainability leaders need to excel at developing relationships with various diverse stakeholders [74]. Employees are key stakeholders in environmental sustainability efforts; therefore, to be effective, leaders need to be able to personalise their approach to each employee to develop strong relationships with the diverse workforce of many organisations. Building individualised relationships with employees will better position leaders to effect environmentally sustainable change through employees.

Taking an individualised approach allows for leaders to leverage individual unique talents. By providing individualised consideration to each employee, leaders can support the individual needs that exist within their team and coach their employees to foster green creativity [2]. There are many different types of PEB, and not every employee will perform every type. Employees’ impressions of PEB may also be limited—leaders may need to show employees all the different types of PEB that are beneficial to environmental sustainability and help connect them to PEBs they will be interested in. Knowing what PEBs are most relevant to individuals will help encourage them. For example, an employee who is very task-oriented and does not see how recycling or conserving energy is relevant to their job might be more engaged with green innovation processes, which requires employees to develop environmentally sustainable products and processes [75,76] as that can more concretely be tied back to their job performance.

Individualisation of approach interacts with another key capability of the proposed ICEER model—communication and influence. The following section will discuss how leaders must develop communication and influence skills to be effective catalysts for employee PEB, but their communication and influence cannot be unilaterally applied. They will need to tailor their approach to their different employees. In this, leadership development needs to prepare leaders to be multi-faceted, identifying their employee’s personal and professional priorities to understand best why they would or would not be motivated to perform PEB and then base their motivation communication strategy and style on that information. This is one of the areas within the ICEER model that points to a gap in the existing research. More research needs to be conducted to understand in what cases the personalisation of communication will best improve employee PEB outcomes.

Regardless of what PEBs they are trying to promote, it is crucial that leaders can pull from multiple leadership styles and skills to individualise their approach to encourage employee PEB. Failed attempts to promote corporate sustainability could also have negative consequences, as managers and employees tire from constant unsuccessful changes and become cynical [77]. Individualising the approach in the early stages of environmental initiatives can help prevent cynicism by incorporating employee feedback into decision-making processes and considering the different individual experiences within the organisation.

**Proposition** **11:** if leaders can individualise their approach while encouraging employee PEB, they will be more effective environmental sustainability leaders;

**Proposition** **12:** when leaders communicate sustainability goals, they must tailor their communication styles to the different departments and employees they work with to achieve the best results.

### 3.11. Communication and Influence Skills: Developing Advocates for Organisational Sustainability

Once leaders have the environmental knowledge and an understanding of their goals as an environmental leader, they need to communicate these goals to their employees and influence their employees to engage with them. Both communication and influence are important: employees need to know what the environmental initiatives are to take part, and since environmental sustainability efforts usually require employees to perform duties outside of their job description [5,52], they need to be influenced to perform these PEBs. Professional communication skills are critical to social, political, and economic functions [78], but they are also multi-faceted. Researchers’ implications frequently call for practitioners to increase open communication across the organisation to achieve desired performance and environmental outcomes [79]. Leadership development programmes should strive to enhance various dimensions of communication skills so leaders can better communicate environmental sustainability challenges and solutions.

Communication skills comprise multiple small social skills that impact how effective messaging and ideas can be. A study examining the role of communication among project managers in sustainable construction project management divided communication skills into 19 different subsets, including listening, motivation, conflict management, writing, and explaining [80]. The framework established in this paper is one example of how communication skills consist of multitudes and how certain forms of communication will be more or less relevant depending on the setting. For example, in construction management, the author noted that reading and writing skills were less valuable than speaking and listening skills [80]. Leadership development needs to train leaders to be good communicators and develop leaders who know the most effective communication forms in each situation and feel comfortable changing their communication style accordingly.

Once leaders can accurately and effectively communicate environmental goals to their employees, they must still influence them to perform PEBs. Influence has been identified as centrally important to leaders’ success in the modern-day [10]. For some employees, being provided information through written or verbal communication about new environmental sustainability initiatives and goals will be enough. This is informational influence, defined as “the influence to accept information obtained from another as evidence about reality” [81] (p.629). Informational influence is most effective when it comes from a trusted source [81]—which again shows the interconnectedness between the key development areas with the ICEER model. Developing ethical leaders will lead to more trust in leaders [63,64]. Increased trust in leaders will make it easier for them to use their environmental knowledge training to exert informational influence on their employees.

Beyond informational influence, we can look to psychology research to see the other types of influence that can play a role in a leader’s ability to increase employee PEB. For example, we can look at the Tripartite Integration Model of Social Influence (TIMSI), which was developed to understand and promote PEB [59]. It is important to note that this model was developed in household settings. However, future testing can validate claims of the TIMSI in the workplace. The TIMSI was developed in social psychology and is primarily focused on social influences through efficacy, identity, and values [59]. The TIMSI is just one example of how identity can influence individuals’ actions.

Within organisational settings, leaders should also note the potential role organisational identity can play in influencing employee PEB. Organisational identity can play an important role in workplace behaviours—particularly those outside of an employee’s role [82]. Therefore, it can be reasoned that if an organisation sets environmental sustainability goals, leaders could leverage organisational identity to increase employee PEB. Leadership development needs to equip leaders to understand what identities are most salient among employees and how those identities could be connected to environmental sustainability efforts.

There are also several cases where normative social influence affects PEB. Normative social influence was defined by Deutsch and Gerald as “an influence to conform with the positive expectations of another” [81] (p. 629). We return to household settings for one example of how effective normative social influence is. Researchers conducted a series of studies among California residents; the first study assessed people’s beliefs about their motivations to conserve energy, and the second study assessed what forms of messaging had the greatest effect on the actual consumption of energy (determined via metered readings). They found that the social norm message encouraged more energy conservation than any other message—despite the perception of social norms’ effectiveness being relatively low [83]. Their findings represent normative social influence as a potentially underutilised motivator of PEB. Future research should aim to replicate these findings in workplace samples. Subsequently, leadership development programmes can educate leaders on utilising normative social influence within their communications to increase their employees’ PEBs.

The combination of communication and influence skills is important for any leader looking to influence their employee’s PEB. Communication skills can enable leaders to convey the goals of environmental sustainability initiatives. Influence skills can allow leaders to increase PEB participation rates among their employees. The ICEER model has called both capabilities out as areas that need more research—there is evidence in the household settings that should be tested in workplaces to determine their effectiveness. By developing these two capabilities in tandem, leadership development can ensure the development of leaders who can promote PEB to all their employees. This will ensure that priorities and changes due to environmental sustainability become salient across all organisational levels.

**Proposition** **13:** increased communication skills will allow leaders to convey organisational environmental sustainability goals clearly;

**Proposition** **14:** employee PEB will increase if leaders can influence employees to adopt sustainable efforts.

## 4. Discussion

This review aims to encourage the expansion of research into the relationship between organisational environmental sustainability and leadership development. It first highlighted why leadership is a worthwhile factor for further investigation, evidencing this with the rise of publications exploring leadership and sustainability in recent years. It then proposes a model that provides research questions to explore and a starting point for practitioner-focused investigations into leadership development interventions to increase organisational environmental sustainability. Past research has focused on leaders’ impact on environmental sustainability. We posit that we need to understand not only whether leaders impact environmental sustainability but also how leaders can impact organisational environmental sustainability.

The connection between leadership development and organisational environmental sustainability needs to be investigated more. However, one essential component seems clear: effective leadership development and commitment to organisational environmental sustainability require a shift in perspective. Organisations’ efforts for environmental sustainability rely on (at least in part) an organisation’s ability to look to the future and take a long-term perspective to solve problems [15,84]. Like investments in environmental sustainability, it may also take time to reap the benefits of leadership development programmes [85]. Leaders will not become change agents for environmental sustainability overnight, and many other social–psychological factors will impact leadership saliency in environmental problem-solving contexts. It is important that practitioners can make long-term cases to decision-makers and set expectations for how long environmental sustainability and leadership development strategies will take to produce tangible benefits. Developing a strong case from the evidence within the scientific literature will be particularly essential for organisations within the Western context that tend to maximise short-term profits over long-term outcomes [15].

Organisations can ensure lasting and impactful change and better understand leadership’s role in organisational environmental sustainability by focusing on how they develop leaders and successfully integrating environmental sustainability into development strategies. Both leadership development and environmental sustainability need a long-term focus [15,84,85]. This similarity presents an opportunity to better understand the costs and benefits of long-term approaches to organisational planning and management. It also presents why research needs to expand our understanding of the relationship between leadership development and environmental sustainability.

The exploration of leadership development and environmental sustainability can yield findings that connect both topics to other areas of investigation within pro-environmental behaviour research and behaviour change as a wider field. In this paper, we chose to shift from an approach siloed in one leadership style to research focused on leadership capabilities. By doing this, we will become more flexible and adaptive. Since leadership development exists within the many contexts within an organisation, a more flexible approach can aim to tackle multiple interventions and strategies within the investigation of leadership development and organisational environmental sustainability.

With this review, we also aim to shift the approach to environmentally sustainable organisations away from strategies that focus purely on external motivation incentives. These external motivation-based interventions can change behaviour in the short term, but there is mixed evidence on the role of financial incentives over time [86]. Due to these mixed results, there is a need for a model that moves away from reliance on financial incentives, highlights the advantages of social–psychological approaches, and is grounded in peer-reviewed research. The ICEER model proposed here puts social–psychological capabilities at the centre of organisational environmental behaviour change efforts. It posits leadership as a unique factor within organisations that can bolster environmental sustainability by developing social and psychological capabilities. By taking a social–psychological approach to environmental sustainability leadership development, our model proposes both a top-down strategy (through the leader’s policy choices) and a bottom-up strategy (through the leader’s ability to encourage employee PEB). This two-pronged approach encourages environmental behaviour change to be impactful, widespread, and sustainable over time. Given the immense challenges posed by climate change and ecological degradation, we need more approaches that will take these holistic approaches to behaviour change.

### 4.1. Theoretical Contributions

This narrative review makes several contributions to the literature. First, it pulls from past research to establish a case for why leadership is an important factor to consider when examining the antecedents of PEB in the workplace. It also makes a case for why researchers and practitioners looking to develop environmentally sustainable leaders should not restrict themselves to one specific leadership style but instead embrace multi-dimensional approaches at the intersection of multiple leadership styles. Finally, the most important theoretical contribution it makes is through the proposal of the ICEER model. The ICEER model provides a framework for exploring how leadership development programmes can impact environmental sustainability. It does this by establishing key capabilities that should be explored more within leadership development and psychology research. This framework creates several avenues for future research, as described below, and provides a foundation for research into how leadership development can impact organisational environmental sustainability.

### 4.2. Practical Implications

This review’s central practical contribution is the ICEER model, which is proposed as a structure practitioners can implement when designing leadership development programmes. The model identifies key capability areas that practitioners can include within their leadership development strategies. Some of the identified capabilities may already be embedded into leadership development programmes, and by highlighting them in the ICEER model, this paper can inform practitioners to place increasing emphasis on those parts of their training. Additionally, by providing takeaways in the form of 14 propositions, this paper has provided an outline for the importance of the various factors within the model that can be presented to a wide range of audiences, allowing environmental and behavioural change practitioners to share the model with people holding varying experiences and knowledge. This review also provides three desired outcome variables, all of which can be measurable and fit various organisational contexts. Measurable outcomes are important within organisational contexts to secure funding for environmental initiatives and obtain support from executive decision-making teams. The combination of key capabilities for leadership development and desired outcome variables makes the ICEER model relevant to both researchers and practitioners.

### 4.3. Limitations and Future Directions

First, this was a narrative review and, therefore, did not encompass all the existing literature; instead, it opted for a more focused approach. This choice was also motivated by the fact that the development of environmental sustainability leadership is still a relatively new topic; therefore, it needed the flexibility to pull research and draw conclusions from a wide variety of papers that do not fit into one set of narrow criteria. As the body of research continues to grow, a more expansive or systematic review approach will be more appropriate. Despite our best efforts to include all relevant papers, the interdisciplinary nature of PEB research means that research is spread widely throughout topics and disciplines, and this review may have omitted some unintentionally. Future research aimed at testing the ICEER model can expand the theoretical grounds of the model as the relationships are explicitly explored in more depth.

We also do come back to a common critique of the existing literature: the prevalence of self-report scales and reliance on correlational research. Arguments utilising the theory of planned behaviour can be made to establish why behavioural intentions and self-report scales have a place in behavioural research. Additionally, there are benefits to self-report scales (such as their low cost), but the evidence base for this model will be strengthened with a mixture of methods. Additionally, correlational studies utilising self-report measures may be good for establishing probable frameworks and illustrating potential effects. Despite this, we must acknowledge that some of the research this model is built on is correlational and does not necessarily mean these are casual relationships, representing one limitation within the current literature that is then reflected in the model. This proposed model still provides a good framework reflective of concepts in the existing literature that can first be verified and modified through empirical testing; then, the model should be tested with behavioural measures for employee PEB, and finally, to increase the ecological validity, the model should be tested experimentally and in practical conditions to determine its effectiveness in leadership development strategies.

Despite the limitations, the development and proposal of the ICEER model provide several pathways for future research. First, the model should be tested through correlation studies and structural equation modelling to confirm the relationships proposed within the model and illustrate the mediation and moderation effects. Once the relationships in the model have been tested, they should be verified in lab settings. Then, studies should be transferred to test the model on working samples across various organisation types and sectors. Importantly, researchers should then transition to testing the model practically by designing leadership development interventions based on the model and testing them in organisational settings. Leadership development takes time [10]; therefore, longitudinal research approaches should be considered after verifying the model via short-term testing.

Finally, although the model proposes that it will be most effective when used holistically, the individual proposals set forth via the model can be utilised within existing research designs and tested within existing leadership development frameworks. Exploration into individual relationships will strengthen our understanding of how these capabilities interact and how the propositions from the ICEER Model can be applied to varying contexts.

## 5. Conclusions

There is a clear interest among researchers and practitioners across disciplines in how organisations can improve their environmental sustainability. One key factor in organisational environmental sustainability is employee PEB. Many factors that influence employee PEB have been identified in the literature, and this review joins the growing number of papers that look at leadership as a central factor in increasing employee PEB. Despite the increasing interest in how leadership can play a role in environmental sustainability, relatively little research still explores how leadership that increases environmental sustainability can be developed. This is an area of research that needs to be expanded and explored. To address this gap, our narrative review draws findings from past research to propose the ICEER model for environmentally sustainable leadership and organisational environmental sustainability.

We recommend that researchers take flexible approaches to investigating leadership development and environmental sustainability that incorporate interdisciplinary practices and knowledge. We also encourage the proliferation of more research focused on developing leaders best suited to influence PEB in communities, organisations, and other settings. With that in mind, the ICEER model strays away from the leadership category and instead focuses on capabilities that leaders of any style or approach can develop. The proposed model consists of 14 propositions highlighting relationships between key capabilities that can be integrated into leadership development to drive three desired outcomes. The ICEER model generates research questions that should be explored and provides a starting structure for practitioners to incorporate environmental sustainability into leadership development. By utilising this proposed model, our goal is so that future researchers and practitioners can work to understand better how leadership development can affect organisational environmental sustainability.

## Data Availability

No new data were created or analysed in this study. Data sharing is not applicable to this article.

## References

[1] Costa A., Mouro C., Duarte A.P. (2022). Waste separation—Who cares? Organizational climate and supervisor support’s role in promoting pro-environmental behaviors in the workplace. Front. Psychol..

[2] Riva F., Magrizos S., Rubel M.R.B. (2021). Investigating the link between managers’ green knowledge and leadership style, and their firms’ environmental performance: The mediation role of green creativity. Bus. Strategy Environ..

[3] Hannah S.T., Avolio B.J., Luthans F., Harms P.D. (2008). Leadership efficacy: Review and future directions. Leadersh. Q..

[4] Farrukh M., Ansari N., Raza A., Wu Y., Wang H. (2022). Fostering employee’s pro-environmental behavior through green transformational leadership, green human resource management and environmental knowledge. Technol. Forecast. Soc. Chang..

[5] Lamm E., Tosti-Kharas J., Williams E.G. (2013). Read This Article, but Don’t Print It. Group Organ. Manag..

[6] Robertson J.L., Barling J. (2013). Greening organizations through leaders’ influence on employees’ pro-environmental behaviors. J. Organ. Behav..

[7] Robertson J.L., Carleton E. (2018). Uncovering How and When Environmental Leadership Affects Employees’ Voluntary Pro-environmental Behavior. J. Leadersh. Organ. Stud..

[8] Wesselink R., Blok V., Ringersma J. (2017). Pro-environmental behaviour in the workplace and the role of managers and organisation. J. Clean. Prod..

[9] Wu J., Zhang W., Peng C., Li J., Zhang S., Cai W., Chen D. (2021). The Trickle-Down Effect of Leaders’ VWGB on Employees’ Pro-Environmental Behaviors: A Moderated Mediation Model. Front. Psychol..

[10] Kets de Vries M.F.R., Korotov K. *Developing Leaders and Leadership Development*. papers.ssrn.com. https://papers.ssrn.com/sol3/papers.cfm?abstract_id=1684001.

[11] Ardichvili A., Dag K.N.O., Manderscheid S. (2016). Leadership Development. Adv. Dev. Hum. Resour..

[12] Boselie P., Dietz G., Boon C. (2005). Commonalities and contradictions in HRM and performance research. Hum. Resour. Manag. J..

[13] Masson-Delmotte V., Zhai P., Pirani A., Connors S.L., Péan C., Chen Y., Goldfarb L., Gomis M.I., Matthews J.B.R., Berger S. (2021). Working Group I Contribution to the Sixth Assessment Report of the Intergovernmental Panel on Climate Change Edited by. www.ipcc.ch.

[14] (2015). “70/1. Transforming our world: The 2030 Agenda for Sustainable Development Transforming our world: The 2030 Agenda for Sustainable Development Preamble. https://sdgs.un.org/2030agenda.

[15] Kantabutra S., Saratun M. (2013). Sustainable leadership: Honeybee practices at Thailand’s oldest university. Int. J. Educ. Manag..

[16] Yuriev A., Boiral O., Francoeur V., Paillé P. (2018). Overcoming the barriers to pro-environmental behaviors in the workplace: A systematic review. J. Clean. Prod..

[17] Kollmuss A., Agyeman J. (2002). Mind the Gap: Why do people act environmentally and what are the barriers to pro-environmental behavior?. Environ. Educ. Res..

[18] Alt E., Spitzeck H. (2016). Improving environmental performance through unit-level organizational citizenship behaviors for the environment: A capability perspective. J. Environ. Manag..

[19] Daily B.F., Bishop J.W., Govindarajulu N. (2009). A conceptual model for organizational citizenship behavior directed toward the environment. Bus. Soc..

[20] Afsar B., Badir Y., Kiani U.S. (2016). Linking spiritual leadership and employee pro-environmental behavior: The influence of workplace spirituality, intrinsic motivation, and environmental passion. J. Environ. Psychol..

[21] Khan A.N., Khan N.A. (2022). The nexuses between transformational leadership and employee green organisational citizenship behaviour: Role of environmental attitude and green dedication. Bus. Strategy Environ..

[22] Kura K.M. (2016). Linking Environmentally Specific Transformational Leadership and Environmental Concern to Green Behaviour at Work. Glob. Bus. Rev..

[23] Blok V., Wesselink R., Studynka O., Kemp R. (2015). Encouraging sustainability in the workplace: A survey on the pro-environmental behaviour of university employees. J. Clean. Prod..

[24] Mouro C., Lomba V., Duarte A. (2021). Pro-Environmental Behaviours at Work: The Interactive Role of Norms and Attitudinal Ambivalence. Sustainability.

[25] Culiberg B., Elgaaied-Gambier L. (2016). Going green to fit in—Understanding the impact of social norms on pro-environmental behaviour, a cross-cultural approach. Int. J. Consum. Stud..

[26] Graves L.M., Sarkis J. (2018). The role of employees’ leadership perceptions, values, and motivation in employees’ provenvironmental behaviors. J. Clean. Prod..

[27] Yang X., Weber A. (2019). Who can improve the environment—Me or the powerful others? An integrative approach to locus of control and pro-environmental behavior in China. Resour. Conserv. Recycl..

[28] Nye M., Hargreaves T. (2010). Exploring the Social Dynamics of Proenvironmental Behavior Change. J. Ind. Ecol..

[29] Khan M.A.S., Jianguo D., Ali M., Saleem S., Usman M. (2019). Interrelations Between Ethical Leadership, Green Psychological Climate, and Organizational Environmental Citizenship Behavior: A Moderated Mediation Model. Front. Psychol..

[30] Li Z., Xue J., Li R., Chen H., Wang T. (2020). Environmentally Specific Transformational Leadership and Employee’s Pro-environmental Behavior: The Mediating Roles of Environmental Passion and Autonomous Motivation. Front. Psychol..

[31] Li M., Gong Z., Gilal F.G., Van Swol L.M., Xu J., Li F. (2021). The Moderating Role of Ethical Leadership on Nurses’ Green Behavior Intentions and Real Green Behavior. Biomed. Res. Int..

[32] Saleem M., Qadeer F., Mahmood F., Han H., Giorgi G., Ariza-Montes A. (2021). Inculcation of Green Behavior in Employees: A Multilevel Moderated Mediation Approach. Int. J. Environ. Res. Public Health.

[33] Whitmarsh L.E., Haggar P., Thomas M. (2018). Waste reduction behaviors at home, at work, and on holiday: What influences behavioral consistency across contexts?. Front. Psychol..

[34] von Borgstede C., Biel A. (2002). Pro-Environmental Behaviour: Situational Barriers and Concern for the Good at Stake. Goteb. Psychol. Rep..

[35] Raineri N., Paillé P. (2016). Linking Corporate Policy and Supervisory Support with Environmental Citizenship Behaviors: The Role of Employee Environmental Beliefs and Commitment. J. Bus. Ethics.

[36] Adekoya O.D., Ajonbadi H.A., Mordi C. (2023). Impact of Green HRM Practices on Employees’ Pro-Environmental Behaviour in the United Kingdom. Global Perspectives on Green HRM.

[37] Warrick D.D. (2011). The Urgent Need for Skilled Transformational Leaders: Integrating Transformational Leadership and Organization Development—ProQuest. J. Leadersh. Account. Ethics.

[38] Paillé P., Mejía-Morelos J.H., Amara N., Norrin H. (2022). Greening the workplace through supervisory behaviors: Assessing what really matters to employees. Int. J. Human. Resour. Manag..

[39] Graves L.M., Sarkis J., Zhu Q. (2013). How transformational leadership and employee motivation combine to predict employee proenvironmental behaviors in China. J. Environ. Psychol..

[40] Cai X., Khan N.A., Egorova O. (2023). Transactional leadership matters in green creative behaviour through workplace learning and green knowledge management: Moderating role of social network sites use. Pers. Rev..

[41] Nduneseokwu C.K., Harder M.K. (2023). Developing environmental transformational leadership with training: Leaders and subordinates environmental behaviour outcomes. J. Clean. Prod..

[42] Odii B.C., Huang Y., Bouvrie N.D., Harder M.K. (2021). Cycles of meaning-making crystallization in the WeValue InSitu process as clear contributions towards transformative learning. J. Clean. Prod..

[43] Mi L., Gan X., Xu T., Long R., Qiao L., Zhu H. (2019). A new perspective to promote organizational citizenship behaviour for the environment: The role of transformational leadershi. J Clean. Prod..

[44] Wu Q., Cherian J., Samad S., Comite U., Hu H., Gunnlaugsson S.B., Oláh J., Sial M.S. (2021). The Role of CSR and Ethical Leadership to Shape Employees’ Pro-Environmental Behavior in the Era of Industry 4.0. A Case of the Banking Sector. Sustainability.

[45] Peng J., Samad S., Comite U., Ahmad N., Han H., Ariza-Montes A., Vega-Muñoz A. (2022). Environmentally Specific Servant Leadership and Employees’ Energy-Specific Pro-Environmental Behavior: Evidence from Healthcare Sector of a Developing Economy. Int. J. Environ. Res. Public Health.

[46] Javed M., Nisar Q.A., Awan A., Nasir U. (2024). Environmentally specific servant leadership and workplace pro-environmental behavior: A dual mediation in context of hotel industry. J. Clean. Prod..

[47] Marques J. (2015). The changed leadership landscape: What matters today. J. Manag. Dev..

[48] Robertson J.L. (2018). The Nature, Measurement and Nomological Network of Environmentally Specific Transformational Leadershi. J. Bus. Ethics.

[49] Lange F. (2023). Behavioral paradigms for studying pro-environmental behavior: A systematic review. Behav. Res. Methods.

[50] Taha A., Hopthrow T., Wu R., Adams N., Brown J., Zoha A., Abbasi Q.H., Imran M.A., Krabicka J. (2021). Identifying the Lack of Energy-Conscious Behaviour in Clinical and Non-Clinical Settings: An NHS Case Study. Electronics.

[51] Wu D.W.L., DiGiacomo A., Kingstone A. (2013). A Sustainable Building Promotes Pro-Environmental Behavior: An Observational Study on Food Disposal. PLoS ONE.

[52] Pinzone M., Guerci M., Lettieri E., Huisingh D. (2019). Effects of ‘green’ training on pro-environmental behaviors and job satisfaction: Evidence from the Italian healthcare sector. J. Clean. Prod..

[53] Ercantan O., Eyupoglu S. (2022). How Do Green Human Resource Management Practices Encourage Employees to Engage in Green Behavior? Perceptions of University Students as Prospective Employees. Sustainability.

[54] Odhiambo G.M., Waiganjo E.W., Simiyu A.N., Kenyatta J. (2023). Green Employee Training—A Remedy for Environmental Behaviour: The Case of Public Universities in Kenya. Eur. J. Bus. Manag. Res..

[55] Schultz P.W. (2002). Knowledge, information, and household recycling: Examining the knowledge-deficit model of behavior change. New Tools for Environmental Protection.

[56] Dickson D. (2005). The case for a ‘deficit model’ of science communication. SciDev. Net.

[57] Nursey-Bray M. (2023). Communicating climate change impacts to Australian coastal and marine communities. Ocean. Coast. Manag..

[58] Cook B.R., Zurita M.d.L.M. (2019). Fulfilling the promise of participation by not resuscitating the deficit model. Glob. Environ. Chang..

[59] Estrada M., Schultz P.W., Silva-Send N., Boudrias M.A. (2017). The Role of Social Influences on Pro-Environment Behaviors in the San Diego Region. J. Urban Health.

[60] Brown M.E., Treviño L.K., Harrison D.A. (2005). Ethical leadership: A social learning perspective for construct development and testing. Organ. Behav. Hum. Decis. Process..

[61] Xuecheng W., Iqbal Q. (2022). Ethical Leadership, Bricolage, and Eco-Innovation in the Chinese Manufacturing Industry: A Multi-Theory Perspective. Sustainability.

[62] Treviño L.K., Brown M., Hartman L. (2003). A qualitative investigation of perceived executive ethical leadership: Perceptions from inside and outside the executive suite. Hum. Relat..

[63] Engelbrecht A.S., Heine G., Mahembe B. (2014). The influence of ethical leadership on trust and work engagement: An exploratory study. SA J. Ind. Psychol..

[64] Brown M.E., Treviño L.K. (2006). Ethical leadership: A review and future directions. Leadersh. Q..

[65] Neubert M.J., Carlson D.S., Kacmar K.M., Roberts J.A., Chonko L.B. (2009). The virtuous influence of ethical leadership behavior: Evidence from the field. J. Bus. Ethics.

[66] Hansen S.D., Alge B.J., Brown M.E., Jackson C.L., Dunford B.B. (2013). Ethical Leadership: Assessing the Value of a Multifoci Social Exchange Perspective. J. Bus. Ethics.

[67] Blau P.M. (1964). Exchange and Power in Social Life.

[68] Mathieu J.E., Hamel K. (1989). A causal model of the antecedents of organizational commitment among professionals and nonprofessionals. J. Vocat. Behav..

[69] Han Z., Wang Q., Yan X. (2019). How Responsible Leadership Motivates Employees to Engage in Organizational Citizenship Behavior for the Environment: A Double-Mediation Model. Sustainability.

[70] Paillé P., Boiral O. (2013). Pro-environmental behavior at work: Construct validity and determinants. J. Environ. Psychol..

[71] Fatoki O. (2019). Hotel Employees’ Pro-Environmental Behaviour: Effect of Leadership Behaviour, Institutional Support and Workplace Spirituality. Sustainability.

[72] Peterlin J. (2016). Incorporation of Sustainability into Leadership Development. Econ. Bus. Rev..

[73] Foster B., Muhammad Z., Yusliza M.Y., Faezah J.N., Johansyah M.D., Yong J.Y., Ul-Haque A., Saputra J., Ramayah T., Fawehinmi O. (2022). Determinants of Pro-Environmental Behaviour in the Workplace. Sustainability.

[74] Iqbal Q., Ahmad N.H., Li Y. (2021). Sustainable Leadership in Frontier Asia Region: Managerial Discretion and Environmental Innovation. Sustainability.

[75] Begum S., Ashfaq M., Xia E., Awan U. (2022). Does green transformational leadership lead to green innovation? The role of green thinking and creative process engagement. Bus. Strategy Environ..

[76] Chen Y.S., Lai S.B., Wen C.T. (2006). The influence of green innovation performance on corporate advantage in Taiwan. J. Bus. Ethics.

[77] Schneider B., Brief A.P., Guzzo R.A. (1996). Creating a climate and culture for sustainable organizational change. Organ. Dyn..

[78] Mullany L. (2020). Rethinking Professional Communication: New Departures for Global Workplace Research.

[79] Iqbal Q., Ahmad N.H., Li Z. (2021). Frugal-based innovation model for sustainable development: Technological and market turbulence. Leadersh. Organ. Dev. J..

[80] Zulch B. Communication skills impact on sustainable and green project management. Proceedings of the World Sustainable Building (SB14) Conference.

[81] Deutsch M., Gerard H.B. (1955). A study of normative and informational social influences upon individual judgment. J. Abnorm. Soc. Psychol..

[82] Lee E.-S., Koo B. (2015). Identifying Organizational Identification as a Basis for Attitudes and Behaviors: A Meta-Analytic Review. Psychol. Bull..

[83] Nolan J.M., Schultz P.W., Cialdini R.B., Goldstein N.J., Griskevicius V. (2008). Normative Social Influence is Underdetected. Pers. Soc. Psychol. Bull..

[84] Avery G.C., Bergsteiner H. (2011). Sustainable leadership practices for enhancing business resilience and performance. Strategy Leadersh..

[85] Amagoh F. (2009). Leadership development and leadership effectiveness. Manag. Decis..

[86] Grilli G., Curtis J. (2021). Encouraging pro-environmental behaviours: A review of methods and approaches. Renew. Sustain. Energy Rev..

